# Sofosbuvir improves HCV‐induced insulin resistance by blocking IRS1 degradation

**DOI:** 10.1002/ctm2.275

**Published:** 2021-01-15

**Authors:** Esther Rey, Javier Ampuero, Francisca Molina‐Jiménez, Patricia Marañón, Yaiza García‐García, Rocío Múñoz‐Hernández, Manuel Romero‐Gómez, Carmelo García‐Monzón, Pedro Majano, Águeda González‐Rodríguez

**Affiliations:** ^1^ Unidad de Investigación Hospital Universitario Santa Cristina Instituto de Investigación Sanitaria del Hospital Universitario de La Princesa Madrid Spain; ^2^ Hospital Universitario Virgen del Rocío Instituto de Biomedicina de Sevilla (IBiS) Sevilla Spain; ^3^ Centro de Investigación Biomédica en Red de Enfermedades Hepáticas y Digestivas (CIBEREHD) Madrid Spain; ^4^ Unidad de Investigación Hospital Universitario de La Princesa Instituto de Investigación Sanitaria del Hospital Universitario de La Princesa Madrid Spain

**Abbreviations**
ALT: alanine aminotransferase
ApoB: apolipoprotein B
AST: aspartate aminotransferase
FIB4: Fibrosis 4 score
GGT: gammaglutamyltransferase; glycated hemoglobin ‐HBa1C‐
HDLc: high density lipoprotein cholesterol
LDLc: low density lipoprotein cholesterol
Tc: total cholesterol
TG: triglycerides
VLDLc: very low density lipoprotein cholesterol

Dear Editor,

In the present study, we have demonstrated that sofosbuvir (SOF) treatment improves systemic insulin resistance in hepatitis C virus (HCV)‐patients, and, for the first time, revealed which molecular mechanisms are involved in SOF effects on the impaired insulin response induced by HCV in hepatocytes.

One of the major concerns in the health care field is the huge number of people affected by chronic HCV infection and its associated complications.^1^ In this regard, chronic HCV infection is associated with hepatic insulin resistance, and an increased risk of diabetes in HCV‐infected patients has been well described.^2^ In fact, the development of these HCV‐related metabolic complications affects the health quality of patients and causes an important burden on medical care. In the last years, the introduction of anti‐HCV regimens based on direct antiviral agents (DAAs) has become a revolutionary advance in the treatment of HCV infection. Among them, SOF, a nucleotide analogue HCV NS5B polymerase inhibitor, was approved by the Food and Drug Administration for the treatment of chronic HCV infection in combination with other antiviral agents since December 2013.^3^ Chronic HCV infection seems to be decreasing with the advent of these new therapies, which is manifesting as a lower burden of cirrhosis and chronic liver failure among waitinglist additions and new transplant recipients.

The obtained results from this study showed that insulin‐resistant HCV‐patients treated with SOF‐based regimens (Table 1) were responders at the end of the treatment, regardless of genotype or degree of liver fibrosis. As expected, a significant decline of serum liver damage markers ‐ AST, ALT, GGT, and bilirubin ‐ concentration was detected in patients at the end of the treatment that remained 1 year later in parallel with a reduction of FIB4 score throughout the study (*P* < .001) (Figure 1A). Moreover, we addressed that SOF‐based treatments could effectively improve the insulin‐resistant state of HCV‐patients. Particularly, a significant reduction of the insulin resistance index HOMA was observed since the end of the treatment (*P* = .0048), being more pronounced 1 year later (*P* < .001), compared with the baseline data (Figure 1B and Figure S1A). Our findings are in agreement with previous data that showed that interferon (IFN)‐free all‐oral DAA regimens improved systemic insulin resistance and blood glucose levels in patients who cleared HCV.^2^

**TABLE 1:** Demographic, anthropometric, virological, and biochemical characteristics of the study population

Feature	Patients (n = 42)
Age (years)	53.5 ± 9.8
Gender (male/female)	29/13 (69%/31%)
BMI (kg/m^2^)	28.5 ± 4.5
Hemoglobin (g/L)	144.7 ± 17.4
Platelets (×10^9^/L)	150 ± 77.3
Albumin (g/dL)	3.9 ± 0.6
INR	1.1 ± 0.2
Creatinine (mg/L)	0.8 ± 0.2
Viral load (log_10_ IU/mL)	6.2 ± 0.7
Fibrosis stage (Fibro scan):	
F0‐ F1	5 (11.9%)
F2	7 (167 %)
F3	8 (19%)
F4	22 (524%)

Data are shown as mean ± standard deviation (SD).

Abbreviations: BMI, body mass index; INR, international normalized ratio.

**FIGURE 1:**
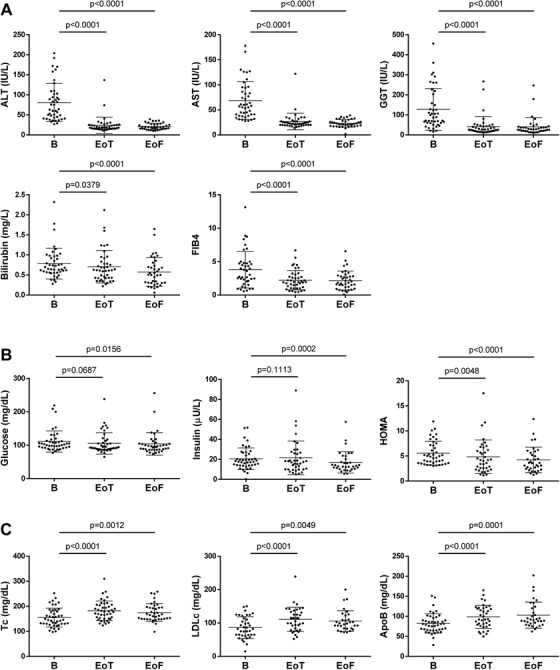
Effects of treatment of chronic HCV patients with SOF‐based regimens on metabolic parameters. Serum levels of ALT, AST, GGT and total bilirubin, and FIB4 score (**A**), serum levels of glucose and insulin, and insulin resistance index, HOMA (**B**), and serum levels of total cholesterol (Tc), LDLc and apolipoprotein B (ApoB) (**C**) from insulin‐resistant HCV‐patients (n = 42) treated with sofosbuvir (SOF)‐based regimens at baseline, at the end of treatment (EoT), and at 1 year after the end of treatment (end of follow up, EoF). Data are presented as mean ± SD and compared by using the Wilcoxon matched‐pairs signed rank test

On the other hand, HCV itself may also impair lipid metabolism since several studies have documented hypocholesterolemia and hypolipidemia, particularly lower levels of total cholesterol (Tc), low density lipoprotein cholesterol (LDLc) and apolipoprotein B (ApoB), the main protein constituent of LDLc and very low density lipoprotein cholesterol (VLDLc), in patients with chronic HCV infection.^2^ Although our study was not designed to clarify the effects of HCV on lipid profile, it has demonstrated that successful HCV clearance resulted in a rebound of circulating Tc, LDLc, and ApoB levels (*P* < .005) (Figure 1C and Figure S1B), according to observations in patients who achieved sustained virologic response with IFN‐based or IFN‐free DAA regimens.^2^, ^4^

In order to explore the possible molecular mechanisms involved in SOF effects on the improvement of insulin resistance observed in patients, we performed an *in vitro* experimental model: HCV replicon‐carrying cells treated with SOF for HCV clearance (Figure 2A and Figure S2) and further stimulated with different doses of insulin. Results obtained from this experimental study have demonstrated that SOF improves the hepatic insulin resistance induced by HCV infection (Figure 2B and Figure S3A). In this regard, it is well known that HCV interferes with the early steps of the insulin signalling cascade, particularly by reducing the expression of the insulin receptor substrates (IRSs), IRS1 and IRS2.^5^ Inactivation of these docking proteins by different mechanisms, such as proteasome‐mediated degradation, has been highly associated with insulin resistance.^6^ Importantly, we observed that HCV induced a reduction of IRS1 protein content in hepatocytes (Figure 2C and Figure S3B), according to previous studies in different cell‐based systems for studying HCV,^5^ and SOF challenge balanced the low protein levels of IRS1 in HCV‐cells (Figure 2C and Figure S3B).

**FIGURE 2:**
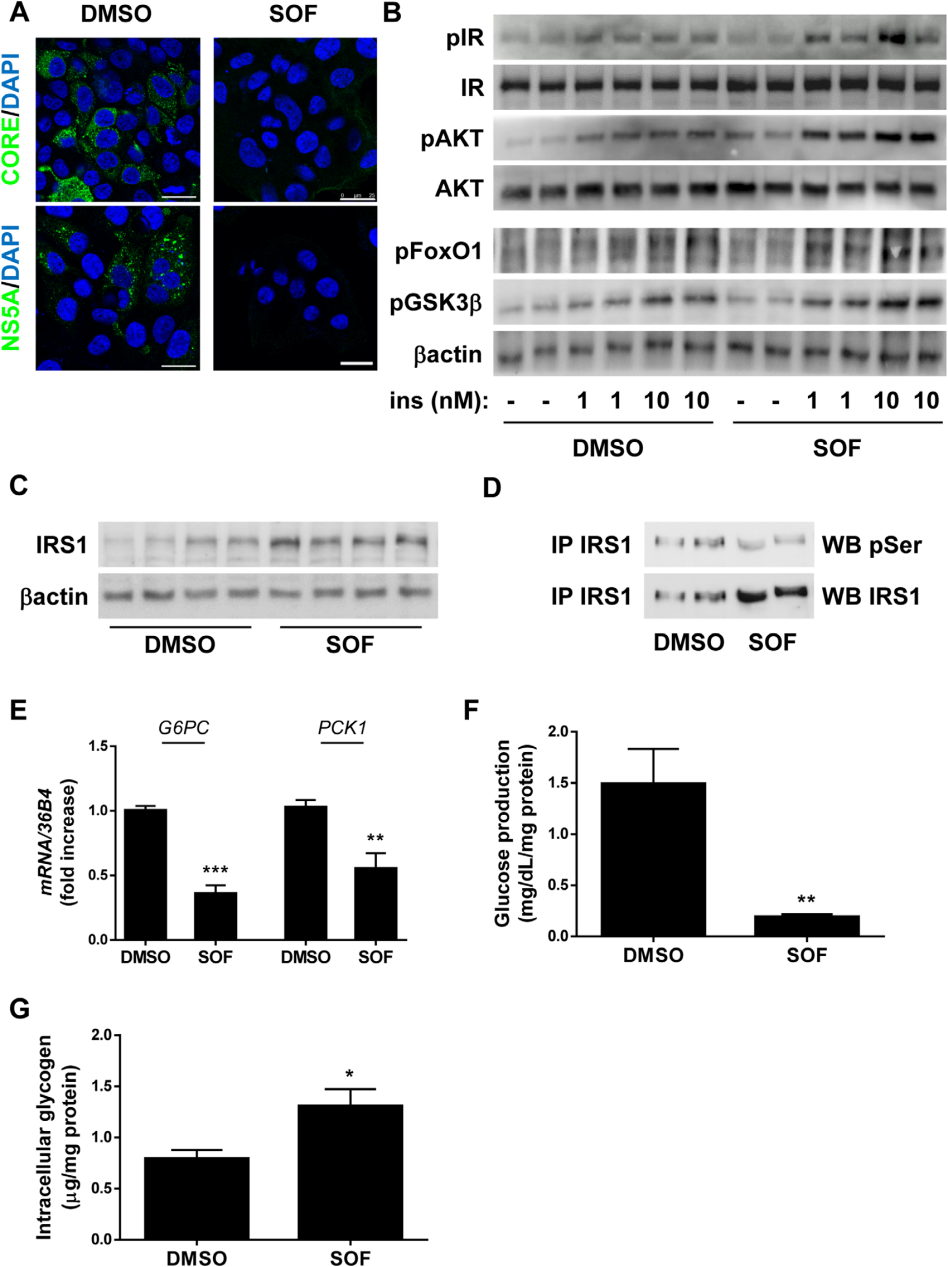
SOF treatment improves insulin response, precluding the transcription of gluconeogenic genes and promoting glycogen synthesis, in hepatocytes expressing full‐length HCV replicons. HCV‐cells were treated with 9‐day vehicle (DMSO) or SOF‐treatment (A, C, D, E, F, and G), and further stimulated with insulin (1 or 10 nM) for 10 minutes (B). A, Representative immunofluorescence images. B, Representative Western blot with antibodies against phospho‐tyrosine1146 insulin receptor (pIR), IR, phospho‐serine473 AKT (pAKT), AKT, phospho‐serine256 FoxO1 (pFoxO1), phospho‐serine9 GSK3β (pGSK3β), and βactin, respectively. C, Representative Western blot with antibodies against IRS1 and βactin, respectively. D, Representative Western blot with antibodies against phospho‐serine (pSer) and IRS1, respectively, after protein immunoprecipitation (IP) with anti‐IRS1 antibody. E, *G6PC* and *PCK1* mRNA levels determined by RT‐qPCR. Data are presented as mean ± SEM and expressed as fold increase and relative to control condition (1). F, Extracellular glucose production. Data are presented as mean ± SEM and expressed as mg/dL normalized to total protein (mg protein). G, Intracellular glycogen content. Data are presented as mean ± SEM and expressed as μg, normalized to total protein (mg protein). *P < .05, **P < .01, and ***P < .005, SOF vs DMSO compared by using the unpaired *t*‐test (n = 3‐4 independent experiments performed in duplicate)

Indeed, we found that serine phosphorylation levels were reduced in HCV‐cells treated with SOF as compared to untreated HCV‐cells (Figure 2D and Figure S3B), which might explain the recovery of IRS1 total protein content after SOF treatment. In this sense, it is well established that HCV core protein induces serine phosphorylation of IRS1 blocking its tyrosine phosphorylation and targets IRS1 for proteasomal degradation.^7^, ^8^ As IRS1 is a critical molecule involved in the transduction of insulin signal from the insulin receptor, its degradation impairs the downstream AKT signalling pathway leading to insulin resistance.^7^ In our study, we observed that SOF treatment greatly improved the response to insulin in AKT phosphorylation and its targets (Foxo1 and GSK3β) in HCV‐hepatocytes compared to DMSO condition (Figure 2B and Figure S3A), reversing the insulin resistance state and, accordingly, the elevated expression of gluconeogenic genes (Figure 2E), the increased glucose production (Figure 2F), and the impairment of glycogen synthesis (Figure 2G) induced by HCV. Other studies have also shown that curing infected cells with IFN treatment partly modifies surrogate markers of insulin resistance such as the upregulated gluconeogenesis.^8^, ^9^ However, to our knowledge, this is the first study showing a reversion of the impaired insulin‐stimulated IR/IRS1/AKT signalling pathway in cured HCV‐cells, which is reflected in a decreased gluconeogenesis and a recover of the glycogen synthesis, giving SOF a key role in the regulation of this pathway that is responsible for its insulin metabolic effects.

Taken together, the results derived from this investigation indicate that SOF improves the impaired insulin response induced by HCV infection. Indeed, this study is especially relevant as it is the first one demonstrating the molecular mechanisms involved in the hepatic insulin sensitization induced by SOF treatment, involving the recovery of IRS1 protein levels as a hallmark of SOF effects, and providing a better understanding of the signalling pathways targeted by SOF in hepatocytes which may offer new insights on the benefits of this DAA.

## CONFLICT OF INTEREST

The authors declare that there is no conflict of interest that could be perceived as prejudicing the impartiality of the research reported.

## Supporting information

Supplementary Information

Supplementary Information

Supplementary Information

Supplementary Information
