# Supplementary figures and images for: Sofosbuvir improves HCV‐induced insulin resistance by blocking IRS1 degradation
Source: Clin Transl Med. 2021 Jan 15;11(1):e275. doi: 10.1002/ctm2.275 (PMC7810262; doi:10.1002/ctm2.275)

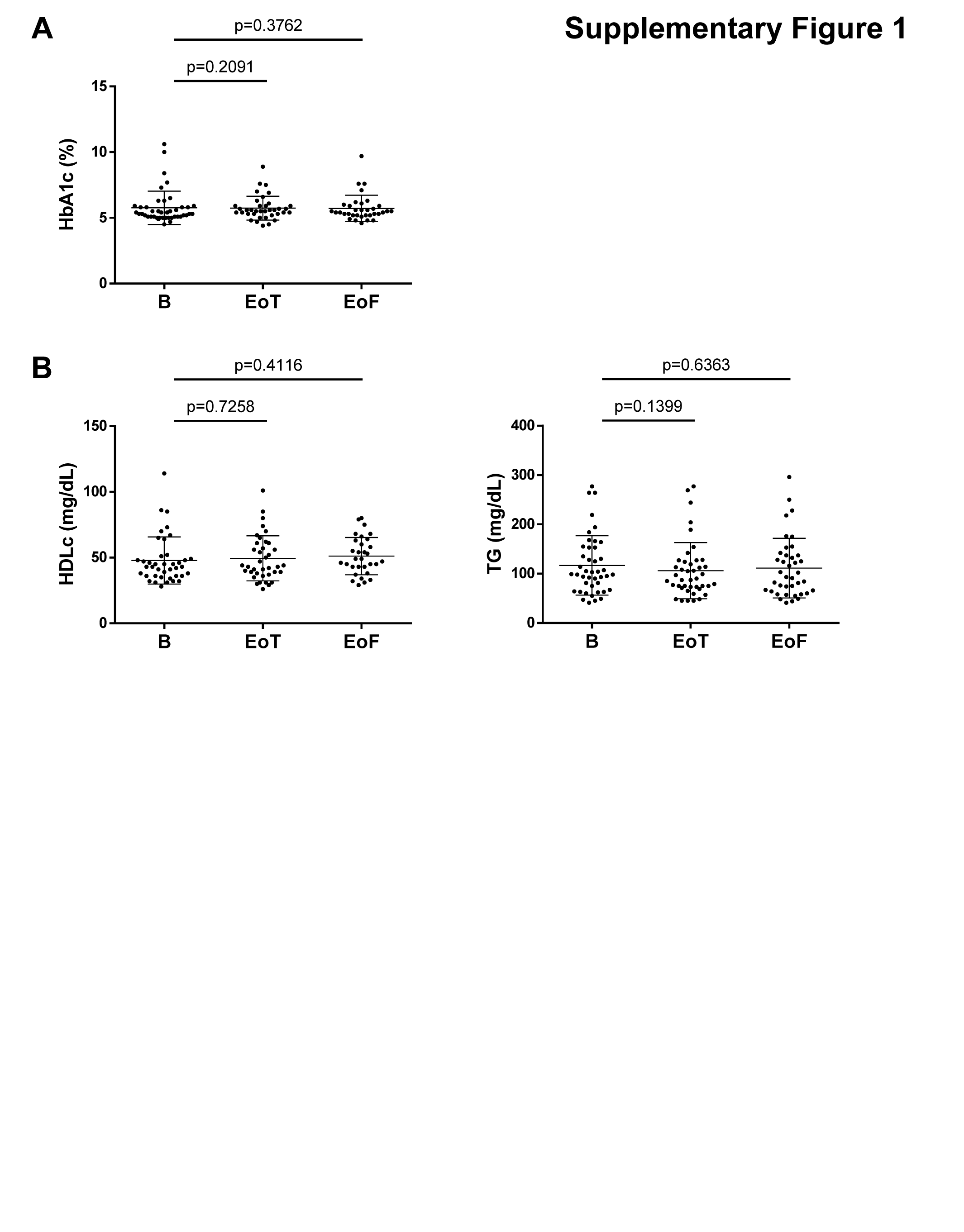

Supplement: Supplementary file 1 — Supplementary Information [file CTM2-11-e275-s001.tif]

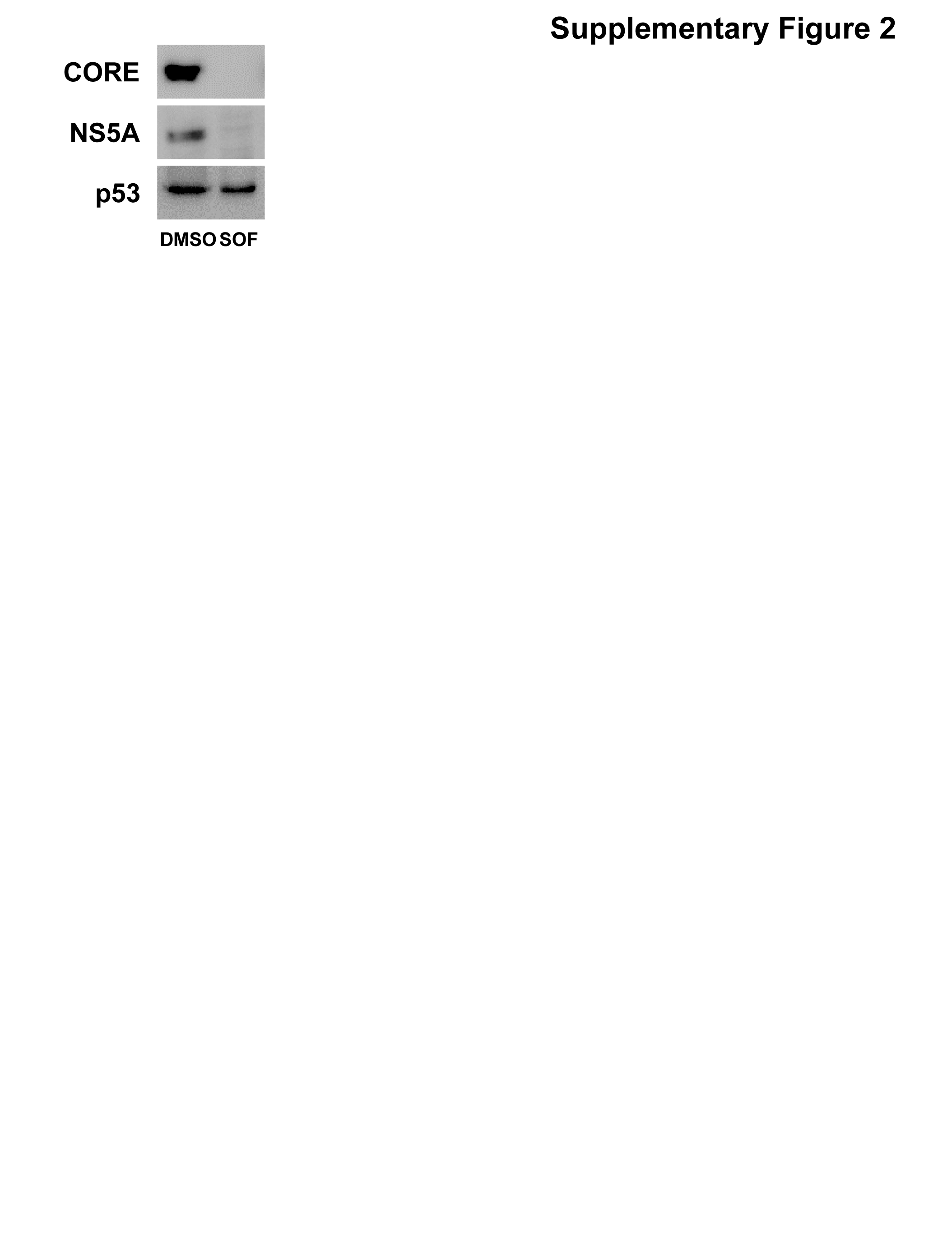

Supplement: Supplementary file 2 — Supplementary Information [file CTM2-11-e275-s002.tif]

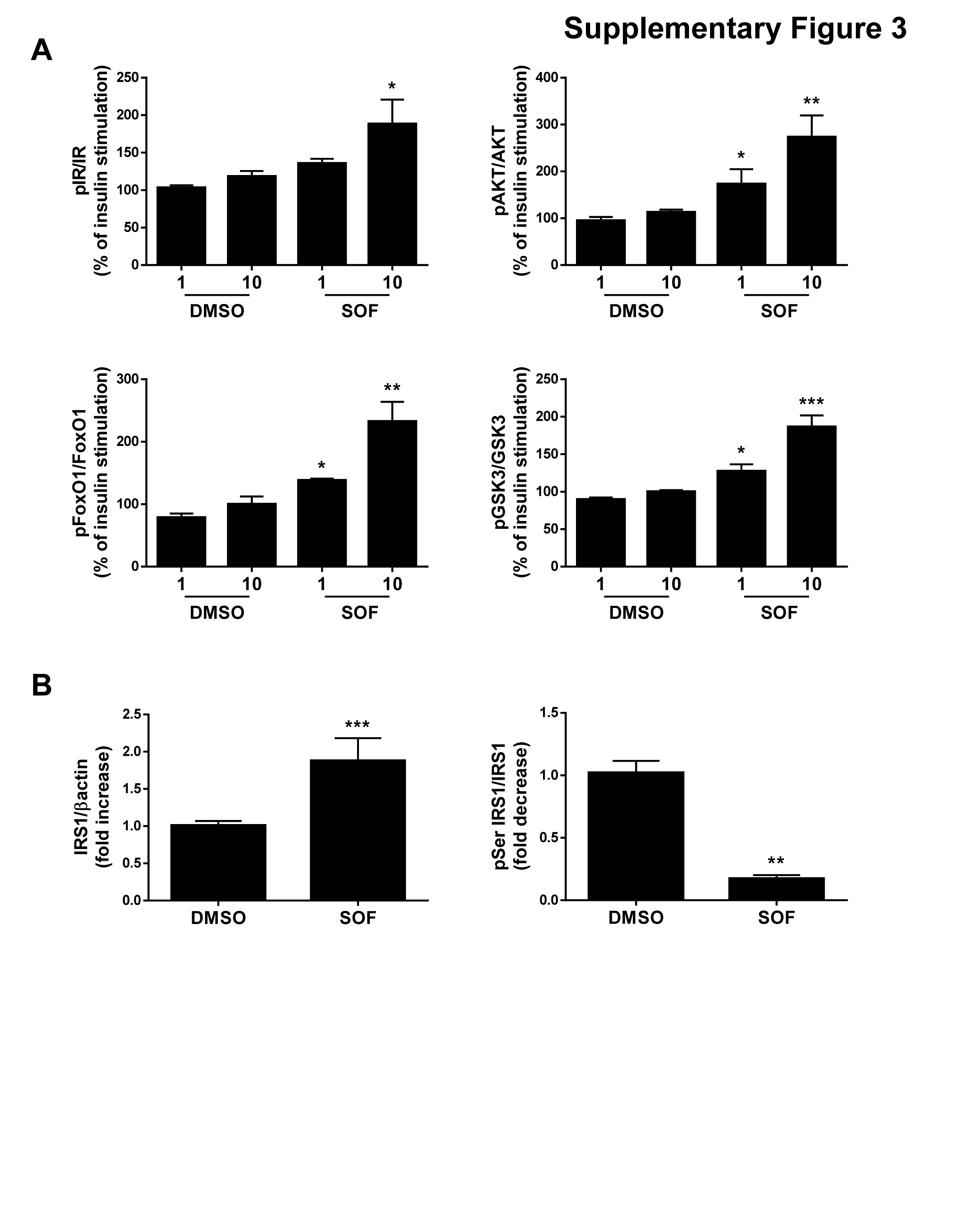

Supplement: Supplementary file 3 — Supplementary Information [file CTM2-11-e275-s003.tif]
