# Supplementary material for: Sofosbuvir improves HCV‐induced insulin resistance by blocking IRS1 degradation
Source: Clin Transl Med. 2021 Jan 15;11(1):e275. doi: 10.1002/ctm2.275 (PMC7810262; doi:10.1002/ctm2.275)
Supplement: Supplementary file 4 — Supplementary Information [file CTM2-11-e275-s004.docx]

**SUPPLEMENTARY MATERIAL**

**SUPPLEMENTARY METHODS**

**Patients.**

The study was conducted in accordance with the principles of the Declaration of Helsinki and International Conference on Harmonization Guidelines for Good Clinical Practice, and was approved by the Ethical Committee of Hospital Universitario Virgen del Rocío (Sevilla, Spain). A written consent was obtained from all the participants.

This retrospective study comprised consecutive patients with HCV-related chronic liver disease that were enrolled at the Digestive Service of the Hospital Universitario Virgen del Rocío (Sevilla, Spain) between January 2015 and February 2017 (n=42). The inclusion criteria were: at least 18 years of age, presence of insulin resistance (insulin resistance index, HOMA > 2.5) at baseline and completion of sofosbuvir (SOF)-based DAA therapy. Patients were included at any stage of liver fibrosis (from F0 to F4), regardless of viral genotype.

Patients were treated with SOF-based regimens according to the physician's choice and received either SOF/daclatasvir (DAC)/ribavirin (RVB) (n=7), SOF/Ledipasvir (LED)/RBV (n=12); SOF/DAC (n=5); SOF/LED (n=16) or SOF/Simeprevir (SIM) (n=2). Duration of treatment ranged from 12 weeks (n=35) to 24 weeks (n=7).

The presence of fibrosis was assessed by non-invasive liver transient elastography (Fibroscan® 402, Echosens, Paris, France): F0-F1, <7 kPa; F2, 7-9,4 kPa; F3, 9,5-13 kPa; F4, >13 kPa.

Detailed data on history and physical examination, along with demographic, anthropometric, virological and biochemical parameters were recorded as follows: age, sex, body mass index (BMI), HCV-RNA value, HCV genotype, previous history of antiviral treatment, and general laboratory tests (aspartate aminotransferase -AST-, alanine aminotransferase -ALT-, gammaglutamyltransferase -GGT-, albumin, bilirubin, creatinine, glucose, insulin, glycated haemoglobin -HBa1C-, total cholesterol -Tc-, low density lipoprotein cholesterol -LDLc-, high density lipoprotein cholesterol -HDLc-, apolipoprotein B -ApoB- and triglycerides -TG-). HOMA was calculated as fasting insulin (μU/mL) × fasting glucose (mg/dL) / 405. Fibrosis 4 (FIB4) score for determination of liver fibrosis was calculated as age (years) × AST (U/L) / [platelets (10^9^/L) × ALT1/2 (U/L)].

For virological, biochemical and metabolic assessments, blood samples were collected at baseline, at the end of treatment (EoT), and at one year after the end of treatment (end of follow up, EoF).

**Cell culture.**

Huh7 cells and their derivatives were grown in Dulbecco's modified Eagle's medium (DMEM) supplemented with 10% fetal calf serum, 2 mM L-glutamine, 50 μg/mL gentamicin, 100 U/mL penicillin, and 100 μg/mL streptomycin, at 37°C in a 5% CO_2_ atmosphere. Huh7 cells expressing full-length genotype 1b HCV replicons (Con1; EMBL database accession number AJ238799) were established as previously described ^[22](#_ENREF_22)^. Briefly, the constructs pI_389_/Core-3’/5.1 and pI_377_/NS3-3’ were linearized with *Sca*I and used as templates for RNA synthesis using the T7 RNA polymerase (Promega, Madison, WI). 20 μg of synthesized RNA were used to electroporate 10^7^ Huh7 cells and 24 hours later 500 μg/mL of geneticin antibiotic (G418) were added in order to select transfected cells. Twice a week culture medium supplemented with G418 was replaced and 4 weeks after transfection the colonies resistant to G418 were isolated and cultured with 100μg/mL of G418

**Antiviral Drugs Treatment.**

Sofosbuvir (SOF, kindly provided by Gilead Science Inc., USA) was reconstituted to 10mM with DMSO. HCV replicon-carrying cells were treated with 10μM SOF for 9 days in the absence of G418 to avoid the death of replicon-eliminated cells. Culture medium was replenished with fresh medium containing SOF or vehicle (DMSO) each 3 days.

**Insulin signalling experiments.**

For the analysis of SOF effects on insulin signalling, HCV replicon-carrying cells were serum starved prior to insulin stimulation (1 or 10 nM, 10 minutes) after DMSO or SOF treatment.

**Immunofluorescence and confocal imaging.**

Cells were fixed with 4% paraformaldehyde in phosphate-buffered saline (PBS) for 10 minutes at room temperature and permeabilized with 0.1% NP-40 in PBS for 10 minutes at room temperature. Then, cells were blocked with TNB (0.1 M Tris-HCl, 0.15 M NaCl, 0.5% blocking reagent -Boehringer Mannheim GmbH, Mannheim, Germany-) for 30 minutes at 37ºC and incubated with the corresponding primary antibodies diluted in TNB for 1 hour at 37ºC: anti-HCV core (clone C7-50, Affinity BioReagents, Goleen, CO) or anti-HCV NS5A (Virostat, Portland, ME). After washing with 0.1% NP-40 in PBS, cells were incubated with Alexa 488 goat anti-mouse (Molecular Probes, Inc., Eugene, OR) for 20 minutes at 37ºC, counterstained with DAPI (Pierce, Rockford, IL) and mounted on DakoCytomation Fluorescent Mounting Medium (DAKO A/S, Glostrup, Denmark). The preparations were analysed with a Leica TCS-SP5 (Leica Microsystems, Heidelberg, Germany) confocal microscope

**Preparation of protein extracts for immunoprecipitation.**

At the end of the experiment, attached cells were scraped off and incubated for 10 minutes on ice with RIPA buffer (50 mM Tris-HCl, pH 7.4, 1% Triton X-100, 0.2% SDS, 1 mM EDTA, 1 mM PMSF and 5 μg/mL leupeptin) to obtain total cell lysates. After protein content determination with Bradford reagent, equals amount of protein (500 μg) were immunoprecipitated at 4ºC with anti-IRS1 antibody (sc-8038, Santa Cruz Biotechnology Inc., Heidelberg, Germany). The immune complexes were collected on sephrose beads (GE17-0120-01, Merck KGaA, Darmstadt, Germany), boiled in Laemmli sample buffer (100mM Tris pH 7.6, 10% (v/v) Glycerol, 6% (p/v) SDS, 0.2% (p/v) Bromophenol blue, 2mM β mercaeptoethanol) and submitted to Western blot analysis.

**Preparation of total protein extracts.**

At the end of the experiment, after washing with PBS, cells were lysed on the plate with 100 μL of Laemmli sample buffer, boiled for 5 minutes and submitted to Western blot analysis.

**Western blot analysis.**

Proteins were separated on a SDS-polyacrylamide gel and transferred onto nitrocellulose membranes (Bio-Rad Inc., Hercules, CA) that were incubated in blocking solution (5% skimmed milk in Tris-buffered saline -TBS- or 3% BSA in TBS) for 1 hour at room temperature. Membranes were then incubated overnight at 4ºC with the corresponding primary antibodies diluted in TBS-T (0.1% Tween-20 in TBS) with 1% skimmed milk or BSA: anti-HCV core (clone C7-50) was from Affinity BioReagents; anti-HCV NS5A was from Virostat; anti-IRS-1 (06-248) was from Merk-Millipore (Darmstadt, Germany); anti-p53 (sc-126), anti-pIR (sc-81499), anti-IR (sc-711), anti-phospho serine (4A3, sc-81516), anti-pAKT (sc-7985-R) and anti-AKT (sc-5298) were from Santa Cruz Biotechnology; anti-pFoxO1 (9461) and anti-pGSK3b (9336) were from Cell Signalling Technology (Boston, MA, USA); anti-βactin (A-5441) was from Sigma-Aldrich Inc. (Madrid, Spain).

Then, membranes were washed three times in TBS-T and incubated with a peroxidase-labeled goat anti-mouse or anti-rabbit IgG (Pierce, Rockford, IL) diluted in TBS-T for 45 minutes at room temperature. After three washes in TBS-T, membrane-bound antibody was visualized with the SuperSignal West Pico Chemiluminiscent Substrate (Pierce). Densitometric analysis of the blots was performed using ImageJ Biological Image Analysis (NIH, Bethesda, MD, USA).

**Gene expression analysis by real-time quantitative PCR (RT-qPCR).**

Total RNA was extracted with TRI Reagent (Vitro, Sevilla, Spain). The cDNA was obtained from 1 μg of RNA by reverse transcription (Reverse Transcription System kit, Promega Inc., Madison, WI, USA). RT-qPCR was carried out in a StepOnePlus™ Real Time PCR System Sequence Detector (Thermo Fisher Scientific Inc., Madrid, Spain) using a SYBR Green kit (Promega Inc.) and d(N)6 random primers that were purchased from Metabion (Planegg, Germany): 5’-TACGTCCTCTTCCCCATCTG-3’ and 5’-TCCCTGGTCCAGTCTCACAA-3’ for glucose 6 phosphatase, 5’-CCAGGCAGTGAGGGAGTTTCT-3’ and 5’-ACTGTGTCTCTTTGCTCTTGG-3’ for phosphoenolpyruvate carboxykinase.

**Analysis of glycogen cellular content.**

After an overnight starvation, cells were washed twice with PBS and resuspended with 200 μL dH2O. After incubation on ice for 10 minutes, samples were boiled at 100ºC for 10 minutes to inactivate enzymes, and centrifuged at 13000 rpm for further 10 minutes. Glycogen content in supernatants was measured with the Glycogen Assay Kit II (ab169558, Abcam, Cambridge, UK).

**Glucose production assay.**

Cells were incubated overnight in serum free DMEM without glucose and supplemented with 2 mM sodium pyruvate. Then, culture media was collected and glucose content was measured using the Glucose Colorimetric Detection Kit (Ref. 15880712, Invitrogen, Thermo Fisher Scientific Inc.)

**Statistical analysis.**

The Shapiro-Wilk test was used to check the normality of the data distribution. Data obtained from the clinical study were presented as mean ± standard deviation (SD). The baseline characteristics of the patients were presented as frequency and percentage. The variables were compared between patients groups (baseline -B-, end of treatment -EoT- and end of follow up -EoF-) by the Wilcoxon matched-pairs signed rank test. Experimental data obtained from the *in vitro* study were presented as mean ± standard error of mean (SEM) and compared by using the unpaired *t* test. All statistical analyses were performed using the IBM SPSS Statistics 24.0 software (SPSS Inc., IBM, Armonk, NY, USA) and the GraphPad Prism 8.0 software (GraphPad Software Inc., San Diego, CA, USA) with two-sided tests, with a p-value of <0.05 considered as statistically significant.

**SUPPLEMENTARY FIGURE LEGENDS**

**Supplementary Figure 1.** Serum levels of HbA1c (**A**), and HDLc and triglycerides (TG) (**B**) from insulin-resistant HCV-patients (n=42) treated with sofosbuvir (SOF)-based regimens at baseline, at the end of treatment (EoT), and at one year after the end of treatment (end of follow up, EoF). Data are presented as mean ± SD and compared by using the Wilcoxon matched-pairs signed rank test.

**Supplementary Figure 2.** HCV-cells were treated with 9-day vehicle (DMSO) or SOF-treatment. Representative Western blot with antibodies against HCV-core, HCV-NS5A and p53, respectively (n=3 independent experiments performed in duplicate).

**Supplementary Figure 3.** HCV-cells were treated with 9-day vehicle (DMSO) or SOF-treatment and further stimulated with insulin (1 or 10 nM) for 10 min. **A**. Quantification of all blots. Data are presented as mean ± SEM and expressed as percentage of insulin stimulation relative to control condition (100%) **B**. Quantification of all blots. Data are presented as mean ± SEM and expressed as fold increase and relative to control condition (1). *p<0.05, **p<0.01 and ***p<0.005, SOF vs. DMSO compared by using the unpaired *t* test (n=3-4 independent experiments performed in duplicate).
